# A Visiting Professorship in Undergraduate Medical Education at the University of Alberta: Reflections on possibilities for medical humanities in China, and elsewhere

**DOI:** 10.15694/mep.2020.000190.1

**Published:** 2020-09-10

**Authors:** Liying Wei, Helly Goez, Tracey Hillier, Pamela Brett-MacLean

**Affiliations:** 1 China Medical University; 2 University of Alberta

**Keywords:** Medical Education, Medical/Health Humanities, Doctor-Patient Relationship, Physicianship, Professional Identity Formation, Curriculum Planning, Humanism, Humanistic Spirit, University of Alberta, China

## Abstract

This article was migrated. The article was marked as recommended.

Enhancing humanities in medical education is a pressing concern in China. Similar to other countries, medical education in China evolved over the past century to emphasize bioscience and technology in treating illness and disease. Increasing recognition of the limitations of biomedical technology led to emergence of the medical humanities in the West in the latter half of the 20 ^th^ century, an interdisciplinary area that has continued to expand and grow. In China and elsewhere, activity in this area developed somewhat later. Ongoing patient-doctor disputes and decline in public trust in the medical profession in China has led many to advocate for enhanced emphasis on humanism and medical humanities. In 2017, the Chinese government introduced new healthcare reforms which included an education and training plan that promotes medical humanities teaching. Global developments have led to a wide variety of models and approaches that may be considered in cultivating medical humanities and humanism in China. With the support of China Medical University in Shenyang, Liaoning Province, PRC, Professor Wei visited the Faculty of Medicine & Dentistry at the University of Alberta through the 2019/20 academic year. This article provides an overview of a wide array of medical humanities teaching and learning opportunities associated with the undergraduate medical education program at the University of Alberta. Professor Wei reflects on possibilities for medical humanities in medical education in China given all she learned and experienced as a visiting professor at the University of Alberta, which may be of interest to others who are also developing new approaches to introducing medical humanities as part of their health professions education program. Additional reflections regarding possibilities for global medical humanities are also offered.

## 1. Introduction

Tremendous advances in biomedical science and technology over the past century have led to many interventions that have proven effective in preventing and treating illness and disease. However, biomedical science and technology-oriented medicine has led many to express concern regarding a fading of “medical humanistic spirit” as reflected in physicians’ “emphasizing the disease but ignoring patients; emphasizing treatment but ignoring patient care; emphasizing lab tests but ignoring patients’ subjective experiences; emphasizing physical recovery but ignoring psychological changes; and emphasizing the use of technology but ignoring ethical and social considerations” ( Song and Tang, 2017, p.129).

A narrow focus on treatment of illness has been associated with a reductionist approach which views the human body as a machine to be fixed when broken. This mechanistic conception of the body, known as the ‘medical model’ or ‘biomedical model’, has led to a bias toward curing disease rather than caring for patients, which should not be the primary end point for healthcare. Indeed, an overriding emphasis on bioscience, and lack of humanity in medicine, has been associated with ongoing patient-doctor tensions and disputes which have led in some incidents to violent outcomes in which Chinese doctors have been killed and injured ( Cai *et al.*, 2019; Lu *et al.*, 2020; Nie *et al.*, 2018; Wang *et al.*, 2017; see Supplemental File 1 for additional text and URLs relevant to this and other sections of this article, cross-referenced by section and paragraph).

In 1946, as a counterpoint to the medical model, the World Health Organization (WHO) introduced a broader, more holistic view defining health as “a state of complete physical, mental and social well-being and not merely the absence of disease or infirmity”. Following from this, Engel’s (1977) introduction of the biopsychosocial model represented an attempt to broaden the conceptualization of health and disease in medicine. Approaching medicine as a healing profession, this model recognizes the need to understand patients’ subjective illness experience, and take into account the complex interaction of biological, psychological, social, and cultural factors associated with health and illness (also see Engel, 1978, 1981; Borrell-Carrió, Suchman and Epstein, 2004; Shorter, 2005).

Engel’s biopsychosocial model emerged as part of a critical social movement in North America in the 1960s and 70s ( Puustinen, Leiman and Viljanen, 2003). Bleakley (2015), referencing the work of Ludmerer (1999), points to the role of the Civil Rights movement and Vietnam War protests in motivating socially responsive educational reforms in the United States of America (USA) that led to introduction of early medical humanities teaching, such as “introduction of professional actors playing ‘standardized’ patients for the purposes of student learning and assessment in simulated clinical contexts” (p.20). An increasingly profit-motivated, technologically-oriented medical profession contributed to a disenchanted, alienated American public, which led to enhanced teaching of medical ethics and history of medicine, and introduction of narrative medicine to help students learn to listen closely and carefully to patients’ stories.

These historical conditions parallel those currently supporting the growth of medical humanities in China. Hou *et al.* (2014) argue that “The imperative for reform is shown by a looming crisis of violence against health workers hypothesized as a result of many factors including deficient educational preparation and harmful profit-driven clinical practices” (p.819). Within this context, Gong *et al.* (2015a) argue that Engel’s model provides a promising framework for supporting the re-integration of a medical humanistic and scientific spirit, stating: “Under the influence of bio-psycho-social medical model, medical humanistic spirit and medical scientific spirit are attached equal importance, which are complementary and mutually reinforcing on the basis of mutual independence. Furthermore, medical humanistic spirit guides the direction for medical scientific spirit.”

## 2. The Global Expansion of the Medical Humanities

Increasing calls for humanizing medicine has led to ongoing growth of the medical humanities (see Bleakley, 2015; Bleakley and Jones, 2014; Chiavaroli, Huang and Monrouxe, 2019; Tang, 2017). A truly global movement, the medical humanities is being introduced all over the world, including Argentina ( Acuña, 2000), Greece ( Batistatou *et al.*, 2010), India ( Shankar, 2016), Japan ( Mori and Nishio, 1996), Korea ( Meng, 2007), Saudi Arabia ( Abdel-Halim and AlKattan, 2012), South Africa ( Hume and Wainwright, 2018), and Turkey ( Elcin *et al.*, 2006), among many other countries.

While no clear definition of the medical humanities exists (see, for example, Campo, 2005; Pattison, 2003), Wu and Chen (2018) have observed Chinese educators active in this area frequently refer to Western definitions, most frequently citing the New York University’s School of Medicine’s (1993) online Literature, Arts and Medicine (LitMed) database definition. Describing the medical humanities as a broad, interdisciplinary field, this definition notes that the arts and humanities provide perspectives and insights “into the human condition, suffering, personhood, and our responsibility to each other”. Similarly, Kirklin (2003) has defined the medical humanities as drawing on “creative and intellectual strengths of diverse disciplines”, while exploring how medical conditions impact individual patients, their families, their communities, and society as a whole, “in pursuit of medical educational goals” (p.1050). Shapiro *et al.*’s (2009) pedagogical definition specifies: 1) use of “methods, concepts, and content from one or more humanities disciplines to investigate illness, pain, disability, suffering, healing, therapeutic relationships, and other aspects of medicine and health care practice”; 2) directed to “teaching health professions students how to better understand and critically reflect on their professions with the intention of becoming more self-aware and humane practitioners”; and 3) “interdisciplinary in theory and practice”, such activities “necessarily nurture collaboration among scholars, healers, and patients” (p.192). More recently, “health humanities” has been proposed as a more encompassing term recognizing inquiry into lived experience of illness and application in other health professions education programs ( Jones *et al.*, 2017; Jones, Wear and Friedman, 2014; also see Lo, Berry and Liping, 2019, p.1; Wu and Chen, 2018, p.2).

Currently, most Western medical schools have integrated the medical humanities as core and/or optional components of the curriculum. A recent survey of 134 American medical schools found over 70% reported medical humanities curriculum content (often integrated into other courses), and 80% offered stand-alone humanities electives ( Klugman, 2018). It was noted that schools with formal humanities programs and structures “have a greater humanities presence in the curriculum” (p.473). Compared to the US, Canada only recently developed a strong medical humanities culture. A survey of Canadian English-language medical schools conducted in 2005/06 identified a broad range of medical humanities teaching offered across all 14 schools, predominantly during the first two years of medical school ( Kidd and Connor, 2008). In 2014/15, a 10-year follow-up survey found evidence of substantive growth in medical humanities teaching, including a wide breadth of arts and humanities educational offerings and approaches, offered through all years of education, across all 17 Canadian medical schools ( Peterkin *et al.*, 2019). In 2015, Bleakley similarly observed “medical humanities components are ... common across UK medical schools” (p.31).

Likewise, in China there is increasing recognition of the need to integrate humanities in medical education to educate doctors who do not just rely on medical instruments and lab results to diagnose and treat the illnesses and complaints of patients they know little or nothing about, other than the most basic clinical information ( Gong *et al.*, 2015a; Gong *et al.*, 2015b; Li *et al.*, 2012; Song and Tang, 2017; Song, Jin and Tang, 2017). Introduction of medical humanities in China began as a response to educational reforms in the 1980s, which led to a variety of curricular innovations in this area, and establishment of the Institute of Medical Humanities at Peking University in 2008. In Taiwan, introduction of medical humanities in the early 2000s followed a recognized need for education reform, partly inspired by a critical review by the USA National Committee on Foreign Medical Education and Accreditation in 1998 ( Chiu, Arrigo and Tsai, 2009; Chou *et al.*, 2012; Wu and Chen, 2018). In 2002, a College of Medical Humanities and Social Sciences was established at Chungshan Medical University in Taichung, Taiwan. Wu and Chen (2018) have described the introduction of the medical humanities at the University of Hong Kong in 2013.

In the West, a growing evidence base suggests the medical humanities can lead to important learning outcomes, such as enhancing observational skills ( Naghshineh *et al.*, 2008) and relational abilities ( Graham *et al.*, 2016; Shapiro, Morrison and Boker, 2004), and professional identity formation ( Clandinin and Cave, 2008; Volpe *et al.*, 2019). Mangione *et al*. (2018) found that exposure to art (visual art, music, literature, theatre, etc.) was associated not only with many positive physician qualities (observational skills, empathy, etc.), but also with reduced burnout. A recent report by the National Academies of Sciences, Engineering and Medicine (2018) concluded that arts and humanities in science, technology, engineering, and medicine leads to positive learning outcomes, such as increased “critical thinking abilities, higher-order thinking and deeper learning, content mastery, problem solving, teamwork and communication skills, improved visuospatial reasoning, and general engagement and enjoyment of learning” (pp.2-3), as well as better grades and higher graduation rates.

What has contributed to the growth of the medical humanities in the East? Vuillermin (2016) suggests that most arguments for including medical humanities in medical education in China are largely similar to those advanced elsewhere, that is, “positivist, biomedical education insufficiently addresses ethical and aesthetic dimensions of medical education and practice” (p.1). Increasing numbers of published reports in China have also described innovative teaching approaches (for example, Chang, Zhou and Zhang, 2014; Gong, Zhang and Hu, 2013; Tsai, 2008; Vuillermin, 2019, 2018; Yin *et al.*, 2016; Wu, 2018), and positive impact of medical humanities teaching on professionalism and humanism ( Fan *et al.*, 2016; Gong *et al.*, 2015b; Guo *et al.*, 2016; Huang *et al.*, 2017; Liao and Wang, 2016; Sherer *et al.*, 2017; Tseng *et al.*, 2016; Wang, Kao and Liao, 2015; Wang *et al.*, 2016; Wong *et al.*, 2012).

## 3. Medical Education and Medical Humanities in China: A Closer Look

Du, Zhang and Adashi (2016) note that, “In the past 100 years, medical education in China has been the subject of multiple changes ... present day policies stand out as pragmatic and as striving for excellence” (p.24). Following the Cultural Revolution, a process of rebuilding medical education has led to the introduction of Western style medical schools across China that offer a variety of options: 3-year secondary medical school programs, to 5- to 8-year programs at comprehensive universities, with the 5-year pathway most common. Medical education programs focus primarily on biomedicine, technology, and clinical medicine. In addition, traditional medicine is included in the curriculum at many Chinese medical schools (see Lam, Wan and Ip, 2006, p.943). Admission to Chinese medical schools does not typically require completion of prerequisite undergraduate coursework, although some schools offer an ‘academic degree exception’ to applicants who have completed a Bachelor degree which allows them to enter accelerated four-year programs. Typically, students who may or may not have selected medicine as their first career choice are accepted into medical school following high school based on their National College Entrance Examination scores.

Hou *et al.* (2014) assert that the breakdown of trust in the healthcare system in China, indicated by ongoing violence against physicians, reflects “a fault line between patient expectations and what doctors and the healthcare system delivers”, and pressing need for “curricular content that would enhance humanistic care that is essential for establishment of rapport between doctors and their patients (e.g., humanities, social sciences, communication skills, ethics, and population health)” (p.826). Based on a thorough review of official government documents, websites, news reports, and published research, they found that the curriculum of most medical schools consists primarily of “didactic lectures requiring rote-memorization”, with a minuscule number of classroom hours assigned “to humanities, social sciences, ethics, public health, psychology, communications skills, and professionalism” (p.824). They also noted lack of exposure to patients, and limited number of humanistic clinical role models as challenges.

A web-based survey of 138 tier-one Chinese medical schools in 2017/18 found that over 2/3rd (67%) offered medical humanities as part of the curriculum ( Kosik *et al.*, 2018). In addition to the compulsory ideological and political theory course (required by all students), the main medical humanities courses included psychology (77%), medical ethics (72%), medical jurisprudence (60%), and doctor-patient communication (49%). Vuillermin (2016) has observed that beginning in the 1980’s through the present, “leading medical schools in China have offered subjects such as philosophy of medicine, medical ethics, medical anthropology, medical aesthetics, health law, and the history of medicine among others; yet only one course is generally compulsory: medical ethics” (p.2).

Du *et al.* (2016) note that longer (5-8 year) medical education programs allow opportunities for exploring other areas of study, “including the humanities” (p.23). Baozhi and Yuhong (2003) have pointed to the need for enhanced integration of basic and clinical science and medical practice, including earlier exposure to patients, and more active approaches to learning. Based on their review of 695 Chinese-language journal articles on medical professionalism (published from 1994-2014), Wang *et al.* (2016) identified growing interest in humanism, and improved physician-patient communication. It was noted that most authors identified “a pressing need to integrate professionalism into medical education through greater availability of humanities courses and participatory learning experiences” (p.4).

Lam *et al.* (2006) have identified a range of innovations that have been introduced in support of medical education reform, including problem-based learning (PBL), as a supplement to didactic teaching, early clinical skills training, use of multimedia technology, and introduction of medical education research and development units. In addition to describing curricular efforts focused on teaching the human side of medicine, and developing the hearts and minds of medical students in China (for example, Shi *et al.*, 2018; Wu *et al.*, 2015; Zhu and Xu, 2014), scholarly effort has also included the study of foreign approaches to medical humanities teaching (for example, Han, 2009; Niu, 2013; Qian *et al.*, 2018; Yin *et al.*, 2002; Xu and Chen, 2007).

Learning about novel teaching approaches can stimulate reflection on accepted assumptions, and openness to appreciating new possibilities for curriculum change. Correspondingly, we have outlined medical/ health humanities (M/HH)teaching and learning at the University of Alberta to both reflect on our own curriculum, and also offer new ideas that might be helpfully imagined for different medical programs, in China and elsewhere.

## 4. Undergraduate Medical Education and M/HH at the University of Alberta

Part of the University of Alberta - a comprehensive, public university, one of the top 100 universities worldwide - the undergraduate MD Program in the Faculty of Medicine & Dentistry (FoMD) is recognized both for its innovative medical curriculum, and excellence in achieving rigorous Canadian and international accreditation standards. It is also recognized for its distinctive medical humanities program, the Arts & Humanities in Health & Medicine (AHHM) (see Bleakley, 2015, p.35). Professor Brett-MacLean, PhD, director of AHHM, and others affiliated with the program, contribute curricular content and a wide variety of elective opportunities to the MD Program, while also contributing to faculty development, co-curricular events, research, and scholarship aimed at supporting the development of well-rounded health professionals through integration of arts, humanities, and social science perspectives in medical education and practice.

The FoMD has many programs for international trainees. Since 2016, the MD Program has organized a Global Summer Student Programwhich offers an opportunity for medical students from China and other countries, to experience different components of the University of Alberta’s medical school curriculum over a four-week period. Tracey Hillier, MD, Helly Goez, MD, and Pamela Brett-MacLean, PhD have all participated in this program, and presented on their work to visiting students.

In August 2019, with the generous funding support of the Chinese Medical University at Shenyang, Liaoning Province, PRC, Professor Liying Wei began a one-year visiting professorship with the MD Program at the University of Alberta. This has supported stimulating discussions between Professor Wei, and Dr. Brett-MacLean (AHHM Director), Dr. Goez, Coordinator of the MD Program’s “Physicianship” course, and Assistant Dean for Equity, Diversity and Inclusiveness, and Dr. Hillier, Associate Dean of Undergraduate Medical Education (2015-2020), as well as other faculty, staff, and students associated with the MD Program. Professor Wei also experienced different approaches to M/HH undergraduate teaching, co-curricular educational offerings, and faculty development opportunities during her visit. In this section, we present an overview of the University of Alberta’s MD Program, including examples of innovative curricular and co-curricular M/HH educational experiences offered.

### 4.1 MD Program: Overall Structure

A four-year post-baccalaureate program, the MD Program provides a rich, interdisciplinary learning environment that supports over 650 medical students in concurrently developing academic knowledge and practical clinical skills. The CanMEDS Physician Competency Framework informs the overall guiding objectives of the medical education program ( Frank and Danoff, 2007; Royal College of Physicians & Surgeons of Canada, 2015). Divided into two phases: pre-clerkship (Year 1 and 2) and clerkship (Year 3 and 4), the curriculum is focused on graduating physicians who are not only qualified (knowledgeable, and technically skilled), but are also compassionate, collaborative, reflective, professional, committed to lifelong learning, and competent in all CanMEDS role areas: Medical Expert, Collaborator, Communicator, Health Advocate, Manager, Professional, and Scholar. Over 1,000 faculty and post-graduate residents contribute over 10,000 hours of planning, course coordination and instruction each year.

In pre-clerkship, an introductory “Foundations” course and integrated organ-based/ systems address basic health sciences (biochemistry, anatomy, pharmacology, medical genetics, etc.) and clinical knowledge and instruction relevant to endocrinology and metabolism, cardiovascular, pulmonary, renal, and musculoskeletal systems, as well as neuroscience, gastroenterology and nutrition, oncology, and psychiatry. In addition to lectures and laboratory instruction, organ-based/ systems courses include both problem-based learning (PBL), called “discovery learning”, and team-based learning. Introduction of course material follows a reasoned progression from basic information to clinical application building on foundational knowledge and skills in a stepwise fashion, with progressively increasing clinical exposure. In Year 3 and 4, clerkship experiences include fully-immersive clinical rotations in family medicine, internal medicine, general and specialty surgery, obstetrics and gynecology, psychiatry, pediatrics, and geriatrics, and clinical learning in rural settings. Throughout the curriculum, students regularly meet with a faculty mentor. This structured approach to personalized mentorship and academic tracking helped to identify students who are struggling academically, ensuring timely remediation and access to support services as needed. In addition, multiple structures have been introduced to enhance professionalism and humanism as part of students’ professional identity formation, including a comprehensive, longitudinal “Physicianship” course, electives, co-curricular learning opportunities, educational scholarship and faculty development, and optional communities of learning.

### 4.2 “Physicianship” Course: M/HH Curriculum Components

Mandatory M/HH curricula in the MD Program is primarily associated with various “curricular threads” offered through the four-year “Physicianship” course, although learning experiences in different systems-based courses also offer M/HH experiences. The “Physicianship” course integrates clinically-relevant knowledge and practice-based skills development to support “practical wisdom.” Incorporating humanistic topics and themes, such as cultural sensitivity, homelessness, organ donation, addictions treatment, sexual and gender minority health, immigrant and refugee health, and Indigenous health, in both “Physicianship” curricular threads as well as organ-based/systems courses, this course supports development of compassion and empathy among medical students as part of their professional identity.

“Physicianship” curriculum threads most closely connected to M/HH include: Communication; Professionalism, Ethics; Social Accountability, Public Health, and Health Systems; Diversity, Cultural Competency/ Safety, Health Equity, and Indigenous Health; and Student Wellness - as well as Physicianship Discussion Groups; Patient Immersion Experience; Longitudinal Clinical Experience; Interprofessional Education; and Academic Service Learning, among others. Additional curriculum threads support development of clinical skills competencies: Nutrition, Physical Examination, Clinical Reasoning, Evidence-Based Medicine, and so on. Sessions require active engagement on the part of students. Formative assessment is used throughout the course; students are required to pass a final integrative exam. Core M/HH-related Physicianship curricular threads - Physicianship Discussion Groups; Longitudinal Clinical Experience; Interprofessional Education; Patient Immersion Experience; Academic Service Learning; “Human Book” Experience; Interpretive Project, and Online Reflection Portfolio - are described in Appendix A.

### 4.3 M/HH Electives

Electives, a mandatory component of the MD Program, provide opportunities for in-depth, individual exploration of different domains relevant to medicine. In pre-clerkship, medical students complete a minimum of 24 elective hours. During clerkship, students complete a minimum of 14 weeks of electives. AHHM organizes a wide range of M/HH electives, and also promotes awareness of M/HH-related electives offered by other departments. Based on a survey of Canadian medical school websites, Lam *et al.* (2015) noted that “With 15 distinct courses geared towards humanities and social sciences, the University of Alberta is the leader among Canadian schools in terms of medical humanities course offerings” (p.36). AHHM currently offers 11 pre-clerkship electives; and three clerkship electives. All are aimed at promoting reflection and enhancing responsiveness to a patient’s experience of health and illness. Electives are pass/fail, based on attendance, active participation, and completion of assignments. See Appendix B for descriptions of five selected AHHM 12-hour, pre-clerkship electives - Introduction to Medical/ Health Humanities; The Healer’s Art: Remembering the Heart of Medicine ( Remen and Rabow, 2005); Communicating Care: A Theatre-based Approach; Shadowing Artists on the Wards: Promoting Patient Centredness; and Transforming Healthcare by Design.

### 4.4 M/HH Co-curricular Opportunities

Relevant M/HH co-curricular learning opportunities are pervasive, including public lectures, exhibitions, performance events, ongoing interest and study groups, conferences, student blogs, research and publication opportunities. In addition to virtual, online opportunities that are open to all, and relevant events organized throughout the University of Alberta, both the AHHM program and the Medical Student Association offer students opportunities for co-curricular M/HH-related exploration and learning. These opportunities are publicized through the AHHM e-Newsand other listservs, calendar and website postings, and posters. Appendix C lists a number of selected AHHM and MSA-related M/HH co-curricular learning opportunities available to medical students at the University of Alberta.

### 4.5 Educational Scholarship/ Faculty Development

Established in 2017, the IDEAS Officesupports health professions education (HPE) scholarship. IDEAS director, Carol Hodgson, PhD, works to promote medical education research capacity by collaborating with FoMD faculty members in developing new research studies by conducting literature reviews, helping to refine research questions and research methods, applying for ethics and, once successful, helping to implement the study, and write up study findings for publication (a recent study evaluated the “Mindfulness” elective offered through the AHHM program). The IDEAS Office also administers a “summer studentship” program on behalf of the FoMD’s Vice-Dean, Education. This program supports students from health professions and other undergraduate degree programs in the FoMD in their exploration of educational scholarship. Students, supervised by a faculty member, receive an honorarium for helping to complete a scholarly project related to health professions education over 1-3 months. Examples of M/HH scholarship completed as summer studentship projects include: 1) development of a customized approach to M/HH “curriculum mapping”, and 2) exploration of “visual pedagogy” in support of individual and collaborative learning in medical school to enhance understanding, reasoning ability, and communication with colleagues, learners, patients, and the public. Outcomes of these projects often lead to the introduction of curricular innovations in the FoMD’s health professions education programs.

Finally, the IDEAS Office “Teaching Scholars Program” (TSP) offers courses in teaching and education to support the development of educational scholars within the FoMD by enhancing knowledge and skills in curriculum development, educational scholarship, and academic leadership. “Fostering Humanism and Professionalism” (TSP 007), based on the curriculum approach and materials developed by Branch *et al.* (2009), was introduced to promote humanistic teaching and role modelling among faculty and residents.

### 4.6 Optional “Communities of Learning”

In 2018, “Communities of Learning” (COL) streams on research and social justice were introduced as an optional component of the MD Program. In 2019, a longitudinal, 4-year M/HH COL was introduced. As intentionally designed groups, learning communities support active collaborative engagement of students and faculty in learning with, and from each other ( Smith *et al.*, 2014). Small group, cohort structures promote relational continuity and a sense of belonging, along with the personal and professional development of students, as students focus on individual and shared areas of interest. Through involvement in COLs, it is hoped that participation will contribute to a sense of purpose and commitment, accomplishment and belonging, and students’ overall well-being.

The M/HH COL requires completion of one of two “gateway learning” elective options (“Introduction to Medical/ Health Humanities,” and/ or “The Healer’s Art”, see 4.3 M/HH Electives described above), plus completion of two additional M/HH electives options, reflective portfolio entries, co-curricular learning experiences, and fulfillment of a leadership initiative, and/or completion of a scholarly project (summer student project or M/HH “directed studies” elective, see Brett-MacLean, 2013). Once students complete their COL requirements, their successful participation is recorded on their Dean’s letter (or final ‘report card’).

## 5. Inspiration for Future Development of Medical Humanities Education in China, and elsewhere

Increasingly, Chinese medical educators are exploring ways humanities can be integrated into medical education. Guo *et al.* (2016) have suggested M/HH should aim to enhance: a spiritual orientation to caring for others, and respecting life, humane care (“beneficence in biomedical research and healthcare”); ability to critically examine medicine through the medical humanities; and, “medical humanities competence, or the ability to take benevolent actions in biomedical research and clinical care.” Nanshan Zhong has similarly argued that medical humanities education in China should not be decorative, directed to only improving “good attitudes” but should be focused on creating a humanistic medicine that conforms to the essential nature of medicine as a healing profession ( Li, 2016).

Based on extensive reading, and her study and experience of many components of the University of Alberta’s MD Program curriculum, electives, and other co-curricular opportunities, along with many conversations with Drs. Brett-MacLean, Goez, and Hillier, Professor Wei has identified a number of ideas regarding the potential of enhanced 1) integration of developmental M/HH teaching and learning, and 2) professional identity formation, and learning, through relationships, while also offering a range of reflections on associated practical considerations for medical education in China (see Table 1).

Professor Wei’s reflections offer rich inspiration for appreciating the many possibilities for ongoing curricular enhancement and renewal. The opportunity to trace and articulate longitudinal M/HH teaching and learning opportunities at the University of Alberta has also helped us to appreciate the possibilities for continuing to evolve our curriculum in relation to pressing educational concerns and societal needs, especially given the current COVID-19 pandemic.

**Table 1.** Professor Wei: Reflections on Possibilities for M/HH in Medical Education in China

**Integrated, Developmental M/HH Teaching and Learning** We should change our traditional thinking and not regard M/HH as a supplementary component of medical education, but make it an integral part to ensure adoption of a humanistic perspective in medicine. Integration of basic and clinical sciences, M/HH teaching, and medical practice (including earlier exposure to patients), and more active approaches to learning (team-based, as well as problem- and case-based teaching methods, scenario simulation, role play, and so on), can promote reflection, foster empathy, and enhance patient care. There are many opportunities for integrating arts and humanities to provide a focus on patients, or the human side of medicine, rather than simply on disease, from pre-clerkship through clinical clerkship rotations, to residency. Introduction of “physicianship” longitudinal curricular themes offers an effective means for teaching the complex set of integrative skills and knowledge medical students need to learn to become compassionate, effective physicians. M/HH teaching and learning opportunities woven into the curriculum can help to ensure deeper understanding, sensitivity, and responsiveness to human dimensions of illness and disease. Many creative possibilities exist, including integrating M/HH into English language classes in Chinese medical schools ( Zhou, 2017). Expanded assessment options should be considered. Besides traditional exams, formative evaluation of students’ reflective writing, art projects, and community service contributions could be applied. Individualized learning options, such as M/HH electives, student clubs, and committees as well as opportunities for organizing and/ or participating in M/HH-related events, such as workshops and symposia, contributes to student engagement and interest. Support of M/HH student initiatives and research in this area can help to develop ongoing leadership and educational scholarship in this area. Together, curricular, co-curricular and other optional M/HH events and activities offer opportunities to deepen understanding of human life and death, and meaning of existence, which can help students internalize care and respect for life. To ensure student motivation to practice medicine is aligned with an understanding of medicine as a caring profession, it may be helpful to consider expanding medical school admission criteria to include such personal qualities such as empathy, moral/ ethical orientation, and passionate commitment to help others. ^a^
**Professional Identity Formation: Learning through Relationships** In-class, theoretical M/HH study and real-life practice should be integrated deeply; it is important to promote development of authentic, caring relationships between students and patients (also see Rutberg, et al., 2017). Faculty mentor role models are important in promoting development of humanism and professionalism among learners. Having students meet regularly with a faculty mentor and engage in small-group discussion can help to achieve this. Medical schools could also collaborate with affiliated hospitals to set up longitudinal M/HH mentorship of students in clinical settings. Cultivating humanistic spirit in medicine requires not only an understanding of patients as people, but also recognizing teachers, students and colleagues as people, with unique personal, cultural, and social backgrounds, as well as varying interests, talents, and experiences, and supporting their development as caring physicians, or “professional identity formation” ( Goldie, 2012). Developing relationships with community organizations and health advocacy groups helps in ensuring a socially accountable curriculum.
**M/HH and Humanism for All: Practical Considerations** Medicine includes a wide range of health professionals who work side by side caring for patients. To improve humanistic care, M/HH education shouldn’t only be directed to developing humanistic qualities of medical students’, but should be part of education for all health professionals, as well as all members of society which is M/HH’s developing trend. Cultivating the medical humanistic spirit requires more than delivery of theoretical knowledge. Practical, programmatic structures, as vehicles for change and transformation, offer a means for coordinated, developmental, socially accountable M/HH curriculum and co-curricular opportunities responsive to the interests and needs of students, faculty members, and the larger community. Recognizing the limited number of M/HH teaching staff, collaborative, interdisciplinary teaching arrangements could be explored. In comprehensive universities, medical teachers, and other health professions educators could cooperate with teachers from arts and humanities departments. Medical teachers at independent medical schools could also reach out to arts and humanities faculty at comprehensive universities. Digital approaches to M/HH learning ( Brett-MacLean, et al., 2018) could potentially help to facilitate such collaborations. ^b^ Introduction of faculty development courses on 1) teaching humanism and professionalism, and 2) M/HH in medical education, more specifically, would also be helpful, as well as opportunities for sharing experiences in introducing M/HH teaching innovations (for example, at a medical education research day).

^a^ *Medical school admissions processes in Canada are directed to identifying a diverse range of capable students who have great promise for being compassionate physicians. All students are required to complete a four-year undergraduate degree; many students accepted into Canadian medical schools have completed Master’s and doctoral degrees prior to medical school. Increasingly, medical students in North America have undergraduate degrees in arts, humanities, and social sciences - in 2007, 15% medical students in the USA had completed a humanities or social sciences undergraduate degree (see Schwartz, et al., 2009).*

^b^ *The COVID-19 pandemic suggests that there are many ways medical education can imaginatively pivot to online teaching approaches, and collegial sharing of digital resources. The challenge remains one of ensuring that this does not result in passive distant learning, but rather aims to promote meaningful, engaged learning.*

## 6. Toward the Future

Currently, there is a feeling of being on the cusp of a new era of medical education in China. Announced in 2017, the Healthy China 2030 project aims to increase the physician workforce, and enhance public satisfaction with the medical profession, through reforms that include ongoing expansion and growth of the medical humanities. While Chinese reports have documented limited, or “very little student exposure to the humanities, social sciences, communication skills, ethics, and population or public health” ( Hou *et al.*, 2014), it is clear that sustained effort is currently being directed to the introduction of M/HH in medical schools to help enhance humanistic care in addition to technical competence among physicians in China. Given increasing support for medical humanities, Yun, Guo and Qian (2017) state, “medical humanities have promise.” By strengthening the medical humanities, Song, Jin and Tang (2017) suggest current educational reforms promise to “foster the humanistic spirits of medical students in order to improve public healthcare in China” (p.366).

Along with the introduction of innovative curriculum change, medical schools in China are also collaborating with other international medical schools as they move forward with educational reform (see Kobza, Dong and Arnaout, 2019; Sherer *et al.*, 2013; She and Liao, 2017). Increasing the openness of education and enhancing educational internationalization, articulated as key strategies in “China’s National Medium- and Long-Term Educational Reform and Development Guideline (2010-2020)” ( Jinqiu, 2012) supported Professor Wei’s visit at the University of Alberta. Given the rapid global expansion of the medical humanities, we appreciate Hooker and Noonan’s (2011) concern regarding unwarranted, wholesale adoption of “Western cultural artefacts, ideas, and *ideals*” (emphasis in original) and need to challenge “uncritical reliance on foundational concepts (such as ‘patient’ or ‘experience’)” as expressive of Western ways of knowing and being in sickness and health (also see Bleakley, Brice and Bligh, 2008). These are concerns that we also share. Recognizing that “what constitutes ‘the medical humanities’ could differ profoundly from place to place” (p.79), we also believe there is a need for ongoing dialogue regarding culture and difference given the global expansion of M/HH (see Naidu, 2020).

We are hopeful that the comprehensive, integrated curriculum developed by the MD Program at the University of Alberta, offers rich inspiration for medical humanities education for others working in this area. We hope that what we have outlined in this paper will provide much substance for discussion regarding different aspects that may prove beneficial to consider adapting in different ways, or may lead to other creative ideas for M/HH curriculum in China and elsewhere. Indeed, many of the medical education innovations introduced at the University of Alberta have been inspired by innovative approaches developed at other medical schools across Canada and other settings, adapted in ways that make sense in relation to the overall MD Program curriculum, while also recognizing the need to fulfill the FoMD’s social accountability mandate in relation to the larger Edmonton community and Northern Alberta region.

Clark (2016) has argued for a Chinese medical humanities informed by a unique constellation of historical events, including rapid three-stage transformation from socialist healthcare, free market reforms in the 1980s, and collectivist counter-reforms in the late 2000s (see Tu, 2019), as well as distinctive medico-cultural traditions and practices, health conditions, and experiences. Many of the articles Wang *et al.* (2016) reviewed considered the role of traditional Confucian and Taoist values, and contemporary political values of the Communist Party in influencing the professional behavior of physicians. Chiavaroli, Huang and Monrouxe (2019) note a re-emergence of Confucianism as an influence on medicine and healthcare in East Asia, primarily with respect to bioethics, in particular as an ethical foundation for elder care ( Koh and Koh, 2008; also see Nie, 2015). With respect to bioethics, Nie (2000) has argued for an interpretive approach to exploring ethical theories and moral practices ranging across a continuum that includes traditional Confucian moral views, socialist cultural ideals, and Western viewpoints. Other similar ideas are being developed and advanced by other medical educators and scholars.

In Canada, Kuper and D’Eon (2011) have argued that insufficient consideration has been given to the required transformation of medical school curriculum to include “relevant, practical and contextualized non-bioscientific knowledge” (p.42), that is, social science and humanities knowledge and scholarship, to support humanistic competencies of physicians. Continuous quality improvement processes have informed curricular change and innovation in the FoMD’s MD Program, based on new directions and “best practices” described in the literature, as well as student feedback and community consultation, while also ensuring alignment with accreditation standards. In 2019, the American Association of Medical Colleges (AAMC, 2020) commissioned a USA/ Canadian research team to assess the role and contributions, and propose potential recommendations for integrating arts and humanities in medical education. It is anticipated that findings from this report will be helpful in guiding M/HH innovations in medical schools across North America over the coming years. A similar national statement regarding the value of, and range of ways humanities and the arts can be integrated in medical education might be considered for China as well.

Heath (2016) has argued that “evidence based medicine tempts us to try to describe people in terms of data from biomedical science: these are not, and will never be, enough. Such evidence is essential but always insufficient for the care of patients. It gives us an alphabet - but as clinicians, we remain unsure of the language.” We propose that an understanding of “the language” of humanistic medicine can best be developed through the introduction of a coherent, integrated M/HH curriculum. We view the medical humanities as critical in supporting the development of medical professionals who are both worthy of, and defenders of public trust. Both in Canada and China, we believe, medical humanities curricular reform should not only be concerned with the personal and professional development of competent physicians who are both ethical and humanistic, but should also be aimed at ensuring improvement in healthcare systems, as well as patient health outcomes, and overall population health.

Describing the humanities (referring to language, history, literature, fine art, philosophy) as “systematic studies of ... human experience ... (using) definite methodologies”, Professor Chan Fai Cheung, former Chair of the Department of Philosophy at the Chinese University of Hong Kong, has argued that “ontologically rooted in the person” humanities disciplines exist to humanize people ( Cheung, 1992). Still, seemingly everywhere, the humanities are under attack with a liberal arts education viewed as a waste of money. Increasing recognition of the ontological contributions of humanities to a liberal arts education, including pre-medical undergraduate degree (where a baccalaureate degree is specified as a medical school admission requirement) and certificate programs, as well as part of undergraduate and postgraduate medical education needs to be reflected in clear support of, *even advocacy for,* humanities teaching and scholarship both within and outside of medical school and other health profession education programs.

Recognizing the importance of the humanities and interdisciplinary thrust of the medical humanities necessitates critical conversations. Together with health profession scholars and educators who can help translate the relevance of arts and humanities perspectives to clinical practice and the culture of biomedicine, faculty experts in different humanities areas can help to ensure the intellectual essence and rigor of their discipline. Of course, the spaces and places in which these inquiries occur also matters in important ways, serving to influence how these inquiries are introduced, to what ends. We suggest that within the context of medicine back and forth dialogue and reflection between medicine and humanities is needed to develop new knowledge, and new ontological, humanistic understandings.

During the past year we have learned much from each other. Just as Chinese medical schools have looked toward the West, there is much that Western medical humanities programs can also learn about the development of medical humanities programs in Asia, and other areas of the world. Within the context of the global COVID-19 pandemic, we believe that M/HH also offers an important lens that can illuminate important issues, questions, and dilemmas, and recognizing our interconnectedness, will hopefully also lead us to exploring new ways we might work together to address concerns related to globalization, climate change, and human health with a view to supporting global well-being, enriched by our diversity and different cultural knowledges. We look forward to continuing to share our knowledge and experiences across cultures, as we all work toward creating a better future for healthcare throughout the world.

## Take Home Messages

• The medical/health humanities (M/HH) provides a powerful lens for learning about the human side of medicine.
• Similar to other countries, there is increasing recognition of the need to integrate M/HH in medical education in China.
• Based on her experience visiting the University of Alberta, Professor Wei describes opportunities for enhanced integration of M/HH to support the development of knowledgeable, technically skilled, and compassionate Chinese physicians.
• To achieve the ontological aims of a humanistic medical education, we need to support humanities education both within and outside medicine.
• Ongoing dialogue regarding global expansion of M/HH is needed.

## Notes On Contributors

**Liying Wei,** **MA,** is an Associate Professor in the School of Fundamental Sciences at Chinese Medical University at Shenyang, Liaoning Province, PRC. Through the 2019-2020 academic year, she was a visiting professor in the Faculty of Medicine & Dentistry at the University of Alberta, Edmonton, Alberta, Canada.

**Helly Goez, MD, FRCPC**, Associate Professor in the Department of Pediatrics, Divisional Director of Pediatric Neurology, MD Program Physicianship Course Coordinator, and Assistant Dean, Equity, Diversity, and Inclusivity for the Faculty of Medicine & Dentistry at the University of Alberta. ORCID ID: https://orcid.org/0000-0003-2474-3741.

**Tracey Hillier, BScN, MD, CCFP, FRCPC, MEd,** Associate Professor, Department of Radiology, Director of the Alberta Institute Wenzhou Medical University, and Associate Dean, Undergraduate Medical Education from 2015-2020, Faculty of Medicine & Dentistry, University of Alberta, is also a member of the Mi’kmaq Qalipu First Nation. ORCID ID: https://orcid.org/0000-0002-4545-7720.

**Pamela Brett-MacLean, PhD** is Associate Professor in the Department of Psychiatry, and Director of the Arts & Humanities in Health & Medicine (AHHM) in the Faculty of Medicine & Dentistry at the University of Alberta. ORCID ID: https://orcid.org/0000-0001-9090-4174.

## Appendices

**Appendix A.** University of Alberta, MD Program - Longitudinal Physicianship Course: M/HH-related Components

Physicianship Discussion Groups	Small groups of ~9-10 students meet regularly with the same humanistic faculty role model across all four years of medical school. Clinical faculty preceptors facilitate reflective discussions on physician identity formation; moral character, professional behavior and responsibilities; patient-centred care; and health and healing. Other topics include: patients’ experience of illness, and its impact on work, family, and social connections; bias and stigma; importance of culture, cultural competency/ humility; ethical reasoning; death and dying; and self-care. (Year 1-4)
Longitudinal Clinical Experience	In pre-clerkship, medical students are individually matched to a community-based family physician. In both years, students spend 7-8 half-days in their preceptor’s clinic where they observe and learn about the doctor-patient relationship, interviewing patients, and physical examination maneuvers. At the beginning of each half-day visit, students share what they are currently learning in medical school with their preceptor, which informs learning objectives and plans for the student’s visit. Physical exams require preceptor approval and supervision, and patient permission. (Year 1-2)
Interprofessional Education	The Interprofessional Learning Pathway, a cross-faculty educational initiative, provides students enrolled in University of Alberta’s health sciences programs (medicine, nursing, dentistry, dental hygiene, radiation therapy, medical laboratory sciences, pharmaceutical sciences, rehabilitation medicine, dietetics, etc.) with progressive interprofessional learning opportunities. At the beginning of their first year, over 1,000 health science students - along with dozens of faculty and staff, patient and family mentors, and professional organizations, including regulatory bodies - participate in an introductory 3-hour launch event. A 1-credit (13-hour) “Foundations of Collaborative Practice” (INTD 403) elective course, along with other 1-credit interprofessional electives (INTD 408/508) offer additional opportunities for further developing foundational understanding and competencies supportive of participatory, coordinated approaches to shared decision-making in healthcare ( CIHC, 2010). (Year 1-2)
Patient Immersion Experience	First-year medical students are matched to a “patient mentor” volunteer, an individual living in the community with a chronic health condition. Initial introductions take place during a large group “meet and greet” event organized by the MD Program. Following this, students visit their patient mentors five times over a two-year period, either at the patient’s home, or at a convenient, accessible public location. Topics discussed include: “Self, Illness, & Family”, “Illness Experience & Empathy”, “Stigma & Illness”, “Breaking Bad News”, and “Lessons Learned”, as well as what it is like to navigate the healthcare system. In second year, students also arrange to attend a medical appointment with their patient mentor as an “accompanying person” (that is, not as a “medical student”). Adapted from a curriculum innovation developed by Kumagai (2009), this experience promotes in-depth understanding into ways illness experience is shaped by factors such as social determinants of health, healthcare beliefs, and family dynamics, which helps students to appreciate patients as whole persons, and the many ways a diagnosis of a chronic health condition can affect different aspects of life. (Year 1-2)
Academic Service Learning	This learning opportunity combines a 20-hour volunteer commitment to community service, with facilitated preparation for, and reflection on this experience. Through relationships established with a variety of non-profit community agencies (such as those supporting immigrants and refugees, people with addictions, people affected by developmental, or intellectual disabilities, sexual and gender minority community members, and homeless community), this curricular thread fosters commitment to social responsibility and caring for diverse patient populations, by helping students gain insight into their personal biases, and better connecting with patients as people. (Year 1)
“Human Book” Experience	Community members who have experienced stereotyping or prejudice accessing healthcare are invited to meet with medical students and speak openly about their experiences (like an “open” book). The aim is to promote intercultural understanding about health disparities through dialogue, with a view to developing rapport, and respectful and affirming exchanges. This session helps students recognize, and develop strategies for mitigating their implicit biases. To date, members of the gay and lesbian community have participated in these sessions. (Year 2)
Interpretive Project	Adapted from a curriculum innovation introduced by Kumagai (see White et al., 2010) and supported by the theory of transmediation ( Siegel, 1995), this activity stimulates exploration of existential, human dimensions of illness while supporting critical reflection on moral aspects relevant to healthcare. In small groups, students explore a question or memorable insight they experienced or learned from their patient mentors (see “Patient Immersion Experience,” above), and “make visible” their new understandings and insights through arts-based media and design projects. This project provides an opportunity for students to embrace a liminal space of uncertainty, and extend their learning through the process of creating a learning artifact to help preserve their commitment to humanistic medicine. A wide range of themes are explored, such as stigma, resilience, and importance of compassionate understanding and support. Examples of projects have included paintings, sculpture, musical compositions, interactive games, etc. The “PIE Interpretive Project” exhibition, organized as part of an end-of-year Patient Appreciation Event, provides an opportunity for patient mentors and their family members to view the students’ projects along with their “artist statements.” Those who experience this exhibition at our end-of-year Patient Appreciation Event have shared they greatly appreciate the creative insights of our second-year medical students. (Year 2)
Reflection Portfolio	As they progress through their third-year clerkship rotations, students write six short reflective narratives about different clinical experiences (error disclosure, ethical dilemmas, breaking bad news, caring for a patient from a marginalized group, conflict resolution, and informed consent), which they submit online. Students reflect on what happened (setting, circumstances, what was said, how others responded, etc.), and describe how what they learned will be helpful to them in their future practice as a doctor. Students later discuss their narrative reflections in their Physicianship Discussion Groups. (Year 3)

**Appendix B.** University of Alberta, MD Program - Selected M/HH-related Pre-Clerkship Electives offered by the AHHM Programa

Introduction to Medical/ Health Humanities	Four-session, small group seminar format; pre-session readings (all students) with supplemental materials, such as an article, essay, poem, etc. assigned to individual students. Discussion focuses on ways arts and humanities contribute to enhanced understanding of lived experience of illness and healthcare within the context of the doctor-patient relationship, healthcare settings and systems. Sessions 1-3: Assigned readings are discussed, followed by student commentary regarding assigned supplemental material. Students also report on health-related media coverage relevant to themes covered. Session 4: For the final session, students prepare and share a verbal, visual, performance, or analytical presentation. Informed by one or more M/HH disciplines, presentations may consider the perspective of a patient, medical student, healthcare practitioner, etc. Guest instructors with backgrounds in different M/HH fields (history of medicine, medical anthropology, philosophy/ethics, literature, art, etc.) are invited to co-facilitate sessions.
The Healer’s Art: Remembering the Heart of Medicine	Five sessions, large and small group discussion, and personal reflection; no required readings or assignments. Developed by Professor Rachel Remen, MD in 1992, The Healer’s Art elective is currently offered throughout the world ( Remen and Rabow, 2005). Sessions include: “Discovering and Nurturing Your Wholeness”, “Sharing Grief and Honoring Loss” (Part 1 and 2), “Allowing Awe in Medicine”, and “Care of the Soul: Service as a Way of Life.” Small groups are facilitated by practicing physicians, who participate as fellow human beings and co-learners. A “discovery” learning approach encourages honest and mutually respectful sharing of experience, beliefs, values, and personal truths. Themes such as sustaining idealism and altruism, medicine as a calling, cultivating compassion, healing loss, and preventing stress and burnout are explored. This elective supports medical students in valuing, enhancing, and preserving the human dimension of healthcare. Faculty preceptors also report a variety of personal and professional benefits, including enhanced commitment to relationship-centred teaching ( Rabow, Newman and Remen, 2014).
Communicating Care: A Theatre-based Approach	Two six-hour workshop sessions, scheduled over a two-day weekend. Theatre practice is founded on awareness of the self as foundational to all aspects of relationship, including communication. Led by professional theatre instructor, Michele Fleiger, experiential theatre exercises support students in learning about themselves and others, and learning to communicate better with patients ( Fleiger, Nagji and Brett-MacLean, 2016). Sequentially introduced, improvisational exercises support students in exploring the “art of communication”, and enhancing their capacity for authentic presence and responsiveness in their interpersonal interactions by recognizing and appreciating multiple perspectives at play in clinical encounters. During debriefing discussions, students reflect on the fundamental value of alert attention (or mindful awareness) for developing meaningful doctor-patient relationships and more effective clinical encounters. This workshop offers a fun and enjoyable approach to enhancing communication skills, particularly in relation to active listening, and non-verbal aspects of communication.
Shadowing Artists on the Wards: Promoting Patient-Centredness	Two one-hour small group introductory and debriefing sessions, eight “shadowing” hours. This elective introduces students to the contributions of artists in hospital settings ( Brett-MacLean *et al.*, 2012). Students shadow literary and visual artists, and musicians who facilitate creative experiences both at the bedside and through group artmaking sessions to help patients translate their anxiety, pain, hopes and dreams into art, writing/ poetry, and music. Students learn about: 1) benefits of the arts in healthcare ( Fancourt and Finn, 2019) in promoting patient and staff well-being, enhancing hospital environments, etc., 2) ways in which creative expression, and listening to patients’ stories can help health practitioners connect with, and relate to patients in more human-centered ways, ^b^ and 3) “best practice” competencies and approaches relevant to arts in healthcare settings (such as patient confidentiality, infection control, communication with healthcare teams, etc.).
Transforming Healthcare by Design	Four three-hour sessions; hands-on, studio-based elective, large and small group format. Led by Patrick von Hauff, professional designer and AHHM affiliate, students explore the relationship between design, health humanities, and the health professions through readings, discussion, and project work. Students from a wide variety of health professions education programs work together in teams of 4-6 members. The first three sessions begin with a 45-minute dialogue with invited speakers from across health and design professions. Following this, students identify a problem they would like to address, which they then explore through multiple perspectives, including interprofessionalism. Through critical discussion, and collaborative design practice, teams develop high-level concepts or proposals for resources, products, environments, services, policies, or systems aimed at positively transforming healthcare. In the final session, student groups present their proposed design solution and invite feedback for further developing their healthcare innovation.

^a^ Other pre-clerkship AHHM electives, not described here, include: Art in Medicine Project; Directed Studies in Medical/ Health Humanities; Introduction to Mindfulness; Spiritual Screening and Assessment: A Communication Skills Workshop; Spirituality and Health: Spiritual Care Shadowing Elective; The Art of Observation: Learning to See (see Elective Catalogue Years 1 & 2 | Faculty of Medicine & Dentistry).

^b^ After shadowing Shirley Serviss, a long-time artist with Artists on the Wards, Professor Wei wrote the following in her reflection journal: *She pushed a trolley decorated with artificial sunflowers and colorful pictures to wards to see patients who made appointments in advance. In it there are some notebooks, storybooks, poem cards and boxes. The trolley was similar to a nurses’ cart, which made me realize that art is also a kind of curing, a spiritual curing. On the ward, Shirley and I talked with patients about poems, stories, books and so on. We communicated freely and the patients enjoyed talking about poems very much. We talked about the poems and kept talking until the patients wanted to rest. During that period of time, I think, patients forgot about their illnesses and pains temporarily, which I can only imagine is helpful to patients. Interacting with patients through different art forms like reading poems, stories, painting, music and so on, helps bring to their minds that they have other identities other than being a patient. Being ill is just one part of life but not the whole. ... One long-term patient shared, “The evenings spent drawing and painting were the only evenings I was free of stress and were usually my only nights of restful sleep.” I learned that artmaking contributes a lot to the care and well-being of patients, medical students, and healthcare staff.*

**Appendix C.** University of Alberta, MD Program - AHHM and MSA-related M/HH Co-Curricular Opportunities

AHHM Speaker Series	The AHHM Speaker Series profiles inspiring work at the intersection of the arts, humanities, health and medicine by noted scholars and practitioners, within and beyond the University of Alberta. In April, 2019, for example, Professor Michael van Manen, MD, PhD, a neonatal pediatrician and phenomenologist, presented on issues and questions he raised in his book *Phenomenology of the Newborn: Life from womb to world* (2018), such as, how should we understand the experience of infants born prematurely who require medical care? What are medical interventions actually like for them? In November, 2019, Judy Rollins, PhD, RN, from Georgetown University and Institute for Integrative Health in Baltimore, Maryland (USA) presented her findings from a multi-year, international study of artwork in hospital environments.
AHHM Occasional Speaker Series	This speaker series provides a forum for medical student presentations. In 2019/20, examples included: “The Conversation that Never Happened” by Gordon Yao (October, 2019) who presented a short film he created to promote awareness and dialogue about mental health challenges experienced by youth of East Asian descent in Canada; as well as “The Fabric of Us” by Adam Devon (November, 2019) who shared his fabric-based, artistic exploration of coping with emotionally challenging situations in medical education; and “The Music of Conversation” by Andrew Kim (January, 2020) which focused on challenges related to authentic communication in healthcare, illustrated through original song lyrics and a musical score. ^a^
Applied Theatre Workshops	David Diamond, founding director of Theatre for Living in Vancouver, British Columbia (Canada), ^b^ has offered workshops in the FoMD since 2012, organized by AHHM. Internationally recognized and respected as an expert in using interactive, theatre-based approaches to explore complex, challenging issues faced by communities, Diamond has helped faculty, staff and students to enhance their understanding of multiple perspectives at play in difficult situations, which has helped to alleviate feelings of alienation and tension, leading to more constructive approaches to working and learning together ( Diamond, 2007). Workshops have explored a wide range of topics, such as team functioning, moral distress, social accountability, health ethics, health and well-being. Although most workshops have been directed to specific groups, departments, centres and units within the FoMD, over the years, many workshops have also been open to the overall university and general public, such as: “Exploring and Responding to Healthcare Challenges Experienced by Persons with Disabilities” (April, 2019); and “Exploring Equity, Diversity, and Inclusion through the Lens of Theatre” (November, 2019).
Calligraphy Workshops	A Clinical Professor in the FoMD, Dr. Steven K.H. Aung is a geriatric, family, and integrative medicine physician, and traditional Chinese medicine (TCM) practitioner who has worked tirelessly to promote the integration of TCM and Western biomedicine in the spirit of a natural, compassionate and artistic approach to health, healing, and wellness. He has received many awards including the Order of Canada in 2006 (Canada’s highest civilian honour), and in 2017, the Dr. Rogers Prize Groundbreaker Award for excellence in complementary and alternative medicine (CAM). Throughout his life, he has created calligraphies and paintings as a field of arts associated with healing, with a view to self-cultivation and promotion of health and well-being. Over the years, he has conducted educational calligraphy workshops as fundraising events in support of integration of art and medicine in the FoMD.
Community Film Screenings	AHHM organizes the “Science in the Cinema” film series to promote understanding and build trust with the community regarding health innovation and research at the university. Held at a local film theatre near the medical school, a variety of films are selected each year: documentary/ biography, drama, comedy, science fiction, and family films. Each film screening is followed by an audience question and answer period with clinicians and health researcher experts from the University of Alberta. Examples of past films (genre/research area) include: “Fantastic Journey” (1966) (Science Fiction/ Nanomedicine); “The Theory of Everything” (2014) (Biography/ Amyotrophic Lateral Sclerosis [ALS]), and “How to Train Your Dragon” (2010) (Family/ Rehabilitation Medicine; Prosthesis). Open to all, with free admission and popcorn, audience numbers range from over 100 to 300 people. Film screenings are consistently well-received by those who attend. In addition to community members, many medical students and faculty members also attend.
Medical Student Association (MSA) ^c^: Student Groups and Initiatives	The Medical Student’s Association (MSA) supports many student-run activities and initiatives. MSA student clubs and groups offer a forum for bringing medical students together who share interest in the arts (such as, music, literary arts, sketching), as well as interest in exploring humanities and social science perspectives on medicine (for example, health systems). In addition, “The Artery” is an annual publication that includes paintings, photography, poetry, musical compositions, and more, showcasing the creativity and artistic passions of students across the FoMD.
MSA: AHHM Student Committee	This student committee connects students across all health professional education programs (nursing, medicine, public health, pharmaceutical sciences, etc.) and arts and humanities disciplines (for example, theatre, design, fine arts, etc.) across the university. Students are supported in bringing forward ideas for activities and events, which have included “Med Student Paint Nights,” art exhibitions, invited talks and presentations. Since 2018, an annual “Create, Collaborate, Connect” symposium event has explored patient experience through art. Invited local artists utilize various art forms, such as spoken word, drama, and visual art, to describe ways healthcare providers accommodated their individual health conditions and concerns, or could have improved on their care. In between performances, participants engage in reflective dialogue in small groups. The aim is to spark honest, unfiltered conversations about tangible ways that patients and healthcare providers can work together towards accountable patient-centered care. In 2019/20, the “Necessary Reflection: Poetry and Medicine” workshop series has provided an opportunity for regular reflection and processing of intense emotions and challenging moments in healthcare. By creating a safe, collaborative environment directed to personal growth in healthcare, these sessions offer an opportunity for students, healthcare providers, and members of the community to appreciate each other’s unique strengths and life experiences.

^a^ All three 4th year medical students travelled to the University of Alberta from Queen’s University in Kingston, Ontario (Canada) to complete a 2-week Directed Studies/ Art in Medicine elective with AHHM. This highly unique clerkship elective attracts medical students from across Canada, as well as from the USA and elsewhere. In April 2020, Gordon Yao’s short video “The Conversation that Never Happened” (5:07 mins) was recognized by an “Art with Impact ‘Short Film’ AWARD” (see https://www.artwithimpact.org/film/conversation-that-never-happened).

^b^ https://theatreforliving.com.

^c^ Medical Students’ Association | Faculty of Medicine & Dentistry.
