# Supplementary material for: A Visiting Professorship in Undergraduate Medical Education at the University of Alberta: Reflections on possibilities for medical humanities in China, and elsewhere
Source: MedEdPublish (2016). 2020 Sep 10;9:190. [Version 1] doi: 10.15694/mep.2020.000190.1 (PMC10699406; doi:10.15694/mep.2020.000190.1)
Supplement: Peer review history

# Reviewer report: Reviewer response for version 1

**DOI:** 10.21956/mep.20151.r31129

**Published:** 2021-02-11

**Authors:** Richard Hays

**Affiliations:** 1 James Cook University

This review has been migrated. The reviewer awarded 4 stars out of 5 As someone with a strong interest in medical humanities I found this an interesting read. I would recommend that all with a similar interest read this. However, I was left with a few questions that the authors might like to consider if a revision is contemplated. The first relates to the purpose and the other to outcomes - was this a one way or two way learning experience? What was the purpose of the visit? I totally support international sabbaticals to broaden experience, but what problem was the visitor trying to address? I have visited formally several medical schools in China and my impression is that medical humanities is either viewed quite differently or regarded as not worthy of much curriculum time (as in most places). On the other hand I had glimpses of really interesting initiatives in art. What was the visitor's experience and with what was he comparing the program at Alberta? Second, what did Alberta learn from China? What might change there? Addressing these issues would make this much more valuable to readers who may be seeking inspiration for their own programs and merit an extra star.

Reviewer Expertise:

NA

No decision status is available

# Reviewer report: Reviewer response for version 1

**DOI:** 10.21956/mep.20151.r31128

**Published:** 2020-12-08

**Authors:** Rong Huang

This review has been migrated. The reviewer awarded 4 stars out of 5 The article provides a comprehensive review of Medical/Health Humanities (M/HH) education around the world, with a particular focus on China. The rich literature could turn out to be very helpful for scholars of medical education as well as other related fields. A comparative perspective further adds to the strength of this article. Prof. Wei is in a unique position to observe and experience two kinds of health systems respectively in China and Canada, which enables her to combine the advantages of both systems while avoiding possible pitfalls. Educators of M/HH would also find the part about reflections and suggestions towards the future M/HH education useful, as some points are quite practical and even ready-to-use, for example, the mentioning of international cooperation and the possibility of developing localized courses based on the existing curriculum as detailed in the article. The authors specially mention the on-going COVID-19 pandemic and re-emphasize the importance of M/HH in this ‘new normal’, so that the article reads both up-to-date and reassuring as it conveys a strong message of consolidation. A curious aspect, however, relates to some of the Chinese authors’ surnames. Neither ‘Baozhi’ nor ‘Yuhong’ sounds like common family names of Chinese people. That said, the article proves to be a timely contribution to the ever-increasingly significant field of M/HH.

Reviewer Expertise:

NA

No decision status is available

# Reviewer report: Reviewer response for version 1

**DOI:** 10.21956/mep.20151.r31130

**Published:** 2020-12-01

**Authors:** Ken Masters

**Affiliations:** 1 Sultan Qaboos University

This review has been migrated. The reviewer awarded 5 stars out of 5 The paper is a fascinating reflective piece based on the experience of a visiting professor from China to Canada, focussing on the possibilities of medical humanities in China.The paper goes much further than one might be led to believe from the title (I think the title sells the paper a little short), and actually gives a very detailed account of medical humanities in both spheres, and grounds the discussion in both personal experience and reference to the relevant literature. The Appendices are also extremely useful, because the rather long paper sometimes threatens to become a little lost in the organisational detail, and readers may be frustrated, asking about the specifics of what is taught; the appendices succinctly and clearly supply this information. The detailed supporting documentation in the supplementary file rounds off the fine paper with further information and references.Overall, a really useful read. I’m sure we all look forward to seeing research initiatives emerging from the lessons learned by all sides.

Reviewer Expertise:

NA

No decision status is available

# Reviewer report: Reviewer response for version 1

**DOI:** 10.21956/mep.20151.r31127

**Published:** 2020-10-15

**Authors:** Dean Parmelee

**Affiliations:** 1 Wright State University

This review has been migrated. The reviewer awarded 4 stars out of 5 Very well-written and comprehensive review of the growing 'medical humanities' domain in medical education. Extensive bibliography is up-to-date and the authors provide crisp summaries in their narrative. The best part of the paper is the history of Chinese medical education and the remarkable attention the government now feels that the humanities needs to be addressed. The type of scholar exchange described is wonderful, and we all hope the current political situation with China abates with an over-due change of leadership in the US.

Reviewer Expertise:

NA

No decision status is available
